# Sodium acetate infusion in critically ill trauma patients for hyperchloremic acidosis

**DOI:** 10.1186/1757-7241-19-24

**Published:** 2011-04-13

**Authors:** Andrew McCague, Mira Dermendjieva, Ryan Hutchinson, David T Wong, Nguyen Dao

**Affiliations:** 1 Department of Surgery, Arrowhead Regional Medical Center, Colton, California; 2 Department of Pharmacy, Arrowhead Regional Medical Center, Colton, California; 3 Western University of Health Sciences, College of Osteopathic Medicine of the Pacific, Pomona, California

## Abstract

**Introduction:**

Sodium acetate has been shown to cause hemodynamic instability when used as a hemodialysis buffer. The pattern of hemodynamic response to injury will be evaluated between those who received sodium acetate and those who did not.

The primary purpose of the study is to analyze the effect of sodium acetate on hemodynamic parameters. Secondarily we looked at the effects on prevention and treatment of hyperchloremic metabolic acidosis.

**Methods:**

The study arm was comprised of patients who had received sodium acetate infusions in place of normal saline between March 2005 and December 2009. A control arm was created based on matching three pre-treatment variables: injury severity score (ISS), pH (+/- 0.03) and base deficit (+/- 3). A retrospective chart review was performed for patients in both arms. Blood pressure, arterial blood gas data and chemistry values were recorded for the time points of -6, -1, 0, 1, 6, 12, 24, 48, and 72 hours from start of sodium acetate infusion. Patients were excluded based on the following criteria: patients who were given sodium bicarbonate within 48 hours of starting sodium acetate, those given sodium acetate as a bolus, non-trauma patients, burn patients, patients who expired within 24 hours of arrival to the ICU, patients diagnosed with rhabdomyolysis and patients whose medical record could not be obtained.

**Results:**

A total of 78 patients were included in the study, 39 in the study arm and 39 in the control arm. There were no statistically significant drops in blood pressure within either group. The median pH between the two groups at the start of infusion was equal. Both groups trended towards normal pH with the study arm improving faster than the control arm. The median serum bicarbonate at start of sodium acetate infusion was 19 mmol/L and 20 mmol/L at time zero for the study and control arms respectively with both trending upward during the study period. Chloride trended up initially in both groups but the study arm began to correct sooner at 24 hours compared to 48 hours for the control arm.

**Conclusion:**

We analyzed the use of sodium acetate as an alternative to normal saline or lactated ringers during resuscitation of critically ill trauma patients at a single center. Our data shows that the hemodynamic profile remained favorable, without evidence of instability at any point during the study period. Normalization of hyperchloremia and metabolic acidosis occurred faster in the patients who received sodium acetate.

## Introduction

One of the most common acid-base disorders seen in trauma patients is metabolic acidosis due to hyperchloremia during resuscitation. In order to prevent and minimize hyperchloremic metabolic acidosis, our institution began to use sodium acetate during resuscitation of trauma patients. As an alternative to normal saline (154 mEq/L chloride) and lactated ringers (109 mEq/L chloride), sodium acetate provides a regimen free of chloride. A source of fixed base, sodium acetate also helps to correct acidosis in patients already acidotic. Acetate is metabolized in muscle to acetyl-CoA which is later used in the TCA cycle where bicarbonate is produced. The use of sodium acetate during resuscitation of trauma patients is presented.

Historically, sodium acetate has been used as a fluid bath for hemodialysis. It became a viable alternative to bicarbonate in the 1960's because of the incompatibility of bicarbonate with solutions containing calcium and magnesium salts [1]. Previous studies have shown that sodium acetate may cause cardiovascular instability in hemodialysis patients. Clinical administration of acetate as part of the hemodialysis treatment is known to be associated with vasodilation and impaired cardiac contractile response [2,3]. Studies on acetate-based dialysates have shown undesirable metabolic side effects such as hypotensive episodes [2,3].

Today, the use of acetate as a hemodialysis buffer is limited. There are few published reports of the use of sodium acetate in non-dialysis patients. In 1978, Kirkendol et al reported that a slow infusion of sodium acetate in dogs did not show a reduction in blood pressure [1]. And as early as 1969, Watten et al showed that sodium acetate solution approached bicarbonate in its ability to restore blood pH and plasma bicarbonate in patients suffering from cholera with metabolic acidosis [4,5]. In 1985, Ekblad et al reported that the continuous infusion of sodium acetate is suitable for the slow correction of metabolic acidosis in premature infants [6]. In spite of the fact that it can be used as an effective buffer in metabolic acidosis, its use has not become widespread [4]. The use of sodium acetate during resuscitation would require a significantly lower acetate load then that previously used for hemodialysis.

The hemodynamic effects of sodium acetate in non-hemodialysis patients are unknown. As of this writing, there are no studies evaluating the hemodynamic effects of sodium acetate infusion in trauma patients. Since 2005, our institution has been using sodium acetate in critically ill trauma patients in an effort to prevent and treat hyperchloremic metabolic acidosis. The pattern of hemodynamic response to injury will be evaluated between those who received sodium acetate and those who did not. The primary purpose of the study is to analyze the effect of sodium acetate on hemodynamic parameters. Secondarily we looked at the effects on prevention and treatment of hyperchloremic metabolic acidosis.

## Methods

Patients who received sodium acetate while in the Surgical Intensive Care Unit (SICU) between March 11^th^, 2005 and December 31^st^, 2009 were selected for the study arm. Sodium acetate was mixed with sterile water to create a chloride free solution. Initial resuscitation was started in the trauma bay with normal saline. Those who remained acidotic after initial crystalloid resuscitation in the trauma bay or operating room and where large volumes of resuscitation were anticipated were started on sodium acetate. Patients were chosen at the discretion of the attending surgical intensivist. Most patients were started on sodium acetate within six hours of arrival. The rate of infusion was chosen based on the patients weight and anticipated fluid volume deficit.

The retrospective review of patient records was approved by our hospital Institutional Review Board (IRB) prior to commencement. Prospective individual informed consent was not obtained as sodium acetate is used routinely at our institution as well as neighboring hospitals.

Patients were excluded based on the following exclusion criteria: patients who were given sodium bicarbonate within 48 hours of starting sodium acetate, those given sodium acetate as a bolus, non-trauma patients, burn patients, patients who expired within 24 hours of arrival to the SICU, patients diagnosed with rhabdomyolysis and patients whose medical record could not be obtained.

Demographic data was collected for each patient including: age, sex, diagnosis, admission and discharge dates, length of stay, reason for SICU admission, Injury Severity Score (ISS) [7], outcome (expired or discharged) and how the sodium acetate was administered. Total acetate load was calculated from rate and duration of infusion. Vital signs and laboratory values at six hours and one hour prior to the initiation of sodium acetate infusion as well as during the hour of infusion, one, six, twelve, twenty-four, forty-eight and seventy-two hours after initiation of infusion. For each time interval, the patients systolic and diastolic blood pressure, mean arterial pressure, arterial blood gas values including pH, lactate and base excess were recorded. From chemistry data the chloride and bicarbonate levels were also recorded. Vital signs were obtained from nursing flow-sheets which records values for each hour. Measurements from arterial lines were used if available, and cuff pressures when not. The chemistry and blood gas data drawn closest to the time interval of interest was chosen with predetermined cut off times.

A control arm was then created using patients admitted between the years 2003 and 2004. During this interval, sodium acetate was not being used and trauma patients were resuscitated with normal saline. All patients in our trauma registry with a matching ISS score to a patient in the study arm were selected. Those patients were then filtered based on the same exclusion criteria outlined above. A single patient was chosen with the best matching pH (+/- 0.03) and base deficit (+/- 3) to the corresponding patient in the acetate group. Time zero was assigned to simulate the start time of the matched patient in the study group. Patients were excluded if they did not have at least three blood gas draws or had less than 36 hours of blood gas data. The same fields were recorded for the control as was for the sodium acetate arm.

A subset analysis was performed on patients who did not receive vasopressors to eliminate its hemodynamic influence on sodium acetate infusion.

### Statistics

Data was analyzed using SPSS (SPSS Inc., Chicago, Il.). Median values were used to limit outliers and minimize variance. Regression values were plotted within SPSS. Wilcoxon signed rank test was used due to the small sample size, non-normal distribution of the study population and repeated measurements performed.

## Results

Initial selection yielded 89 patients for the sodium acetate arm and 1107 patients for the control pool. After evaluation for exclusion criteria and matching, there were 39 patients eligible for the study group and a corresponding 39 patients were matched from the remaining control pool. Six patients could not be matched for ISS and the next closest ISS was used.

Demographic data is presented in Table 1. Age and sex between study and control groups were not statistically significant. The mean ISS between the two groups were 32.6 and 31.1 in the study and control groups respectively. Vasopressors were used in 43.6% of the study group and 30.7% of control group which was not statistically different (p-value = 0.23). Mean infusion time of sodium acetate was 38.1 hours. The estimated acetate loads range from 5.02 to 182.1 total grams.

**Table 1:** Demographic data

	Sodium Acetate	Control	P-Value
**N**	39	39	

**Mean Age**	43.4 (21-86)	39.9 (15-90)	0.24

**Sex M/F (% Males)**	28/11 (71.8%)	30/9 (76.9%)	0.53

**Mean ISS Score**	32.6 (4-75)	31.1 (14-50)	

**Patients on Vasopressors**	17 (43.6%)	12 (30.7%)	0.23

**Mean Infusion Time (hours)**	38.1 (4-167)		

**Median Acetate Load (grams)**	31.8 (5.02 - 182.1)		

**Type of Trauma**			

MVC	15 (38.5%)	27 (69.2%)	0.01

Auto vs peds/cycle	13 (33.3%)	5 (12.8%)	0.05

GSW/Stab	4 (10.3%)	7 (17.9%)	0.26

Other	7 (17.9%)	0	0.01

**Figure 1:**
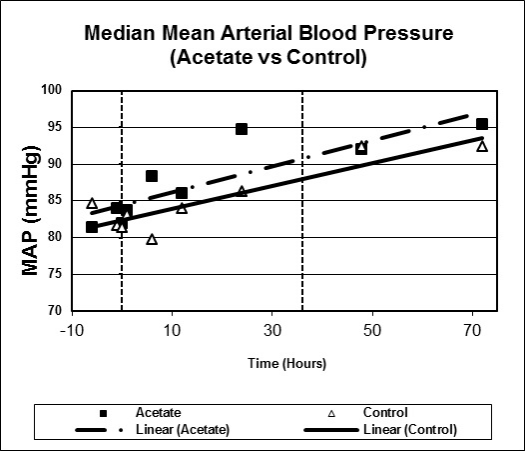
**Scatter plot of median mean arterial pressures, study versus control arms**. Start time (0 hours) and mean infusion time (38 hours) noted with vertical lines. Regression lines plotted with p-value 0.257.

**Figure 2:**
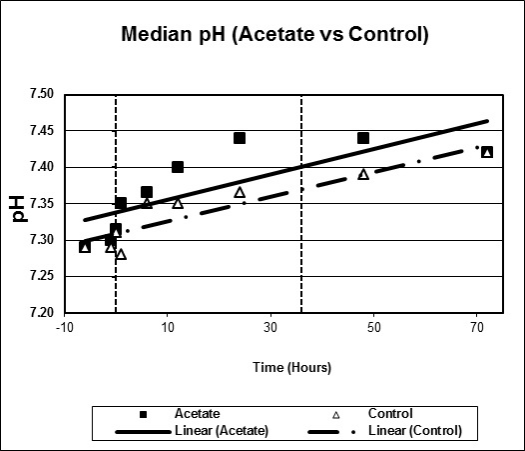
**Scatter plot of median pH, study versus control arms**. Start time (0 hours) and mean infusion time (38 hours) noted with vertical lines. Regression lines plotted with p-value 0.175.

**Figure 3:**
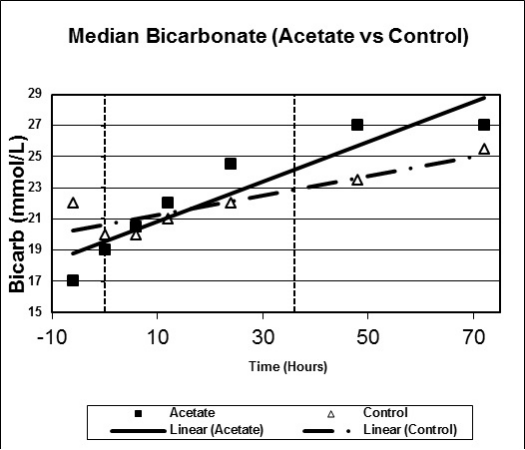
**Scatter plot of median bicarbonate, study versus control arms**. Start time (0 hours) and mean infusion time (38 hours) noted with vertical lines. Regression lines plotted with p-value 0.93.

**Figure 4:**
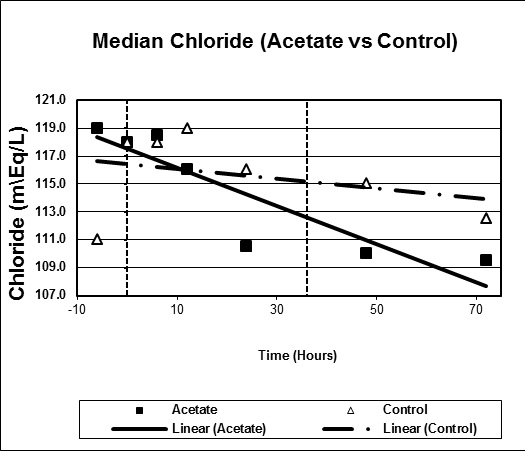
**Scatter plot of median chloride, study versus control arms**. Start time (0 hours) and mean infusion time (38 hours) noted with vertical lines. Regression lines plotted with p-value 0.618.

**Figure 5:**
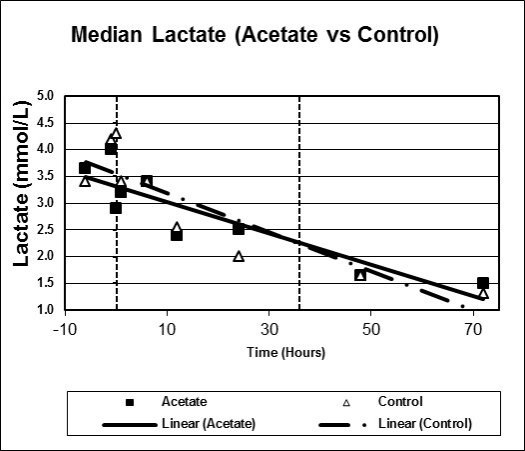
**Scatter plot of median lactate, study versus control arms**. Start time (0 hours) and mean infusion time (38 hours) noted with vertical lines. Regression lines plotted with p-value 0.792.

**Figure 6:**
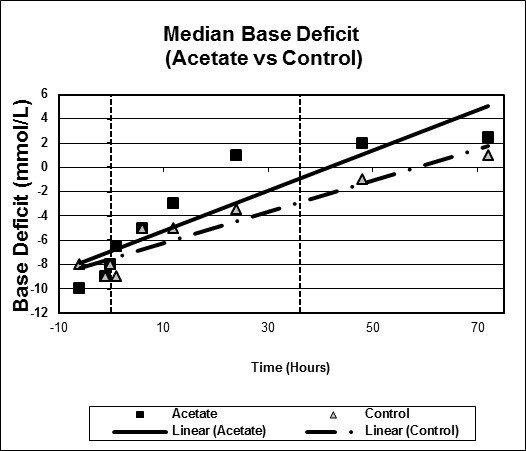
**Scatter plot of median base deficit, study versus control arms**. Start time (0 hours) and mean infusion time (38 hours) noted with vertical lines. Regression lines plotted with p-value 0.498.

Figures 1, 2, 3, 4, 5, 6 plot median values for each parameter collected for both the study and control arms. Mean arterial pressure (MAP) trended up in both the study and control arms. The control arm had an initial drop in MAP which improved with time. Regression line comparison did not show a statistically significant difference between the two groups with a p-value of 0.257. There were no clinically significant changes in MAP during the time intervals studied. The median pH at time zero was 7.32 and 7.31 in the sodium acetate and control arms, respectively. There was normalization of the pH over time in both groups. The study group showed a more rapid improvement in pH but both groups normalized by the end of the study period. When comparing regression lines a p-value of 0.175 suggested no statistical difference. The median serum bicarbonate level at time zero was 19 mmol/L in the study arm and 20 mmol/L in the control arm. The study arm patients began with much lower bicarbonate levels prior to time zero. The plots show a more rapid improvement of bicarbonate in the study group as compared to the control. Comparison of regression lines did not show a statistically significant change between the two groups with a p-value of 0.93. Patients in both groups were hyperchloremic prior to the start of infusion. The median serum chloride concentration was 118 mEq/L in both the study and control arms at time zero. Both trended toward normal over time. The p-value was 0.618 when comparing regression lines. Median lactate levels at time zero were 2.9 mmol/L in the study group and 4.3 mmol/L in the control group. In both groups lactate trended down. Regression lines were not significantly different with a p-value of 0.792. Both groups began with high base deficits which trended toward normal during the study period. Regression lines were not significantly different with a p-value of 0.498.

Within the sodium acetate group there were 17 patients who received vasopressors and 12 patients in the control group. Vasopressors consisted almost exclusively of norepinephrine, for those who did not respond, vasopressin was also used. In order to limit vasopressor influence, a subset-analysis was performed on those patients who did not receive vasopressors comparing the study arm and control arm. Each time interval was compared to time zero using Wilcoxon signed rank test, p-values are reported (Table 2). A statistically significant rise in MAP values starting at 24 hours post start of acetate infusion for the study group was found. For the control group, there were no statistically significant changes as compared to time zero for the duration of the study period. Neither group showed drops in hemodynamic variables.

**Table 2:** Comparison of MAP at time zero to each subsequent interval for patients who did not receive vasopressors.

	Time (Hours)					
	**0 to 1**	**0 to 6**	**0 to 12**	**0 to 24**	**0 to 48**	**0 to 72**

**Acetate**	0.896	0.183	0.408	**0.011**	**0.017**	**0.004**

**Control**	0.926	0.881	0.983	0.632	0.058	0.177

P-values listed (statistical significance defined by <0.05).

## Discussion

In this study, we show that sodium acetate infusion in the critically ill patient is a viable alternative in the prevention of hyperchloremic metabolic acidosis. To date, there have been no published studies evaluating the use of sodium acetate in critically ill trauma patients. Previously published studies have suggested that sodium acetate can cause hemodynamic instability in dialysis patients. We have used sodium acetate for five years in our SICU without experiencing clinically evident hemodynamic instability. This study looks at the effects of sodium acetate on hemodynamic stability and hyperchloremia.

Our study of the use of sodium acetate in trauma patients did not reveal the hemodynamic instability as seen in previous studies where sodium acetate is used in dialysis patients. Sodium acetate improved acidosis faster, without drops in hemodynamic parameters. The use of a chloride free solution prevents the accumulation of chloride and development of hyperchloremic metabolic acidosis.

Though there are limitations to our study we feel that the use of sodium acetate is a viable alternative to normal saline during resuscitation of trauma patients. The small sample size in our study limits the clinical relevance of our results and may lead to a higher risk of Type I statistical error. We are limited by the number of available patients during this time period. There is also a lack of standardization when choosing which patients received sodium acetate. Though initially designed to avoid a selection bias, from preferentially choosing sicker patients to receive sodium acetate, use of historical cohorts from different time periods may actually introduce selection bias as the resuscitation strategies may have changed over time. Similarly, excluding patients who expired within 24 hours may have eliminated those patients who needed sodium acetate the most. Being a retrospective study, measuring acetate and sodium load for each patient is not possible, patients are exposed to multiple sources of each. An exact measurement will help control for this variation. The use of ISS inherently has limitations, patients with similar ISS may have very different injuries and different outcomes. The use of vasopressors amongst a large portion of our patients may reduce the clinical relevance of the results. Vasopressors not only affect blood pressure, they can also affect pH. However, a subset analysis was performed with patients not on vasopressors showed no adverse hemodynamic instability. Future work will include a prospective randomized study which will help to overcome the significant limitations of a retrospective study. Currently we can only comment on trends within the data and are not able to make absolute conclusions.

Our analysis of the use of sodium acetate during resuscitation of trauma patients has shown that sodium acetate is an alternative to normal saline and lactated ringers in prevention of hyperchloremic metabolic acidosis. Previous reports of hemodynamic instability are not validated in this study.

## Conclusion

We analyzed the use of sodium acetate in critically ill trauma patients at a single center, during a four-year period. Our data shows that the hemodynamic profile remained favorable, without evidence of instability at any point during the study period regardless of vasopressor use. Normalization of hyperchloremia and metabolic acidosis occurred faster in this group compared to control patients.

## Competing interests

The authors declare that they have no competing interests.

## Authors' contributions

All authors made significant contributions leading to the final manuscript. AM authored the manuscript, helped design the study, performed data collection and statistical analysis. MD designed the study, performed data collection and helped author manuscript. RH helped with study design and performed data collection. DW helped conceive the study, participated in study design and helped revise and edit the manuscript. ND helped conceive and coordinate the study as well as and data mining. All authors have read and approved manuscript.
